# Estimating cutoff values for diagnostic tests to achieve target specificity using extreme value theory

**DOI:** 10.1186/s12874-023-02139-5

**Published:** 2024-02-08

**Authors:** Sierra Pugh, Bailey K. Fosdick, Mary Nehring, Emily N. Gallichotte, Sue VandeWoude, Ander Wilson

**Affiliations:** 1 Department of Statistics, Colorado State University, 102 Statistics Building, Fort Collins, 80523 Colorado USA; 2 Department of Biostatistics and Informatics, Colorado School of Public Health, Aurora, Colorado USA; 3 Department of Microbiology, Immunology, and Pathology, Colorado State University, Fort Collins, Colorado USA

**Keywords:** Antibody test, Cut point, Extreme value theory, Serology, Threshold

## Abstract

**Background:**

Rapidly developing tests for emerging diseases is critical for early disease monitoring. In the early stages of an epidemic, when low prevalences are expected, high specificity tests are desired to avoid numerous false positives. Selecting a cutoff to classify positive and negative test results that has the desired operating characteristics, such as specificity, is challenging for new tests because of limited validation data with known disease status. While there is ample statistical literature on estimating quantiles of a distribution, there is limited evidence on estimating extreme quantiles from limited validation data and the resulting test characteristics in the disease testing context.

**Methods:**

We propose using extreme value theory to select a cutoff with predetermined specificity by fitting a Pareto distribution to the upper tail of the negative controls. We compared this method to five previously proposed cutoff selection methods in a data analysis and simulation study. We analyzed COVID-19 enzyme linked immunosorbent assay antibody test results from long-term care facilities and skilled nursing staff in Colorado between May and December of 2020.

**Results:**

We found the extreme value approach had minimal bias when targeting a specificity of 0.995. Using the empirical quantile of the negative controls performed well when targeting a specificity of 0.95. The higher target specificity is preferred for overall test accuracy when prevalence is low, whereas the lower target specificity is preferred when prevalence is higher and resulted in less variable prevalence estimation.

**Discussion:**

While commonly used, the normal based methods showed considerable bias compared to the empirical and extreme value theory-based methods.

**Conclusions:**

When determining disease testing cutoffs from small training data samples, we recommend using the extreme value based-methods when targeting a high specificity and the empirical quantile when targeting a lower specificity.

**Supplementary Information:**

The online version contains supplementary material available at 10.1186/s12874-023-02139-5.

## Introduction

When faced with an emerging infectious disease outbreak, it is imperative to rapidly develop diagnostic tests to determine individual disease status and estimate community prevalence. Both individual- and community-level information is necessary to target public health interventions and deploy medical resources. In addition to designing tests that accurately measure biological samples for evidence of disease (e.g., antibodies), a critical challenge is how to classify quantitative test results as positive or negative. Therefore, a threshold, based on controls with known disease status, must be selected to determine positive and negative test results.

Estimating cutoffs for newly developed tests provides unique challenges. First, tests can show little separation in the distributions for positive and negative controls. The threshold can be chosen to target a particular sensitivity or specificity, but not both. Second, many early tests have a limited number of controls with known disease status. For example, a study found that of 47 coronavirus disease 2019 (COVID-19) antibody tests used in developing countries, the majority had fewer than 200 negative controls and some had as few as 31 [1]. Thus, estimating the cutoff that will have the desired sensitivity or specificity must be done from limited data.

This raises two important questions. First, what sensitivity or specificity should be targeted? Second, how to best estimate a cutoff value for the target sensitivity or specificity? For emerging diseases, we expect the prevalence to be low. Thus, to optimize the number of tests with the correct result, we should prioritize correctly identifying negative results and consequently have a high specificity [2, 3]. For this reason, the Centers for Disease Control and Prevention (CDC) recommended high specificity, such as 0.995, for tests developed in the early part of the COVID-19 pandemic [4]. To achieve a target specificity, researchers commonly use the same quantile of the negative controls distribution as a cutoff. Hence, the objective is to estimate the 0.995 quantile of a likely skewed distribution from limited training data. Two common approaches to estimating a quantile of the negative controls are to use the empirical quantile or use the quantiles of a parametric distribution, such as normal or lognormal, fitted to the data [3, 5–7]. However, these methods have not been specifically evaluated for selecting cutoffs of rapidly developed tests for emerging diseases and the resulting test characteristics.

We provide two contributions to the literature. First, we propose using methods from extreme value theory literature to estimate a cutoff for a desired target specificity. Our proposed approach is to fit a generalized Pareto distribution to the upper tail of the negative control data [8]. This approach has been broadly used to estimate extreme values of events such as rainfall [9], air pollution exposures [10], and stock prices [11], among other applications, but has never been applied to cutoff selection. Second, we compare commonly used methods and the proposed extreme value-based approach, for estimating the cutoffs of emerging disease tests through a simulation study and data application. We compare cutoff estimation methods based on their accuracy in achieving a target specificity, individual tests, and estimating community prevalence. We also compare the impact of target specificities on these outcomes. In our data analysis, we focus on enzyme linked immunosorbent assay (ELISA) antibody test data collected during the first year of the COVID-19 pandemic. However, the methods proposed are general and can be applied to data from any test. In our simulation study, we demonstrated the extreme value method had the least bias for estimating a cutoff for a high target specificity and that lower target specificities are easier to estimate and may perform better when the objective is estimating prevalence.

## Methods

### Data

We used two data sources in our analysis. The training dataset contained blood samples from staff at long-term care facilities in Colorado, USA, sampled between June and December of 2020. A total of 226 staff members underwent up to five tests each, resulting in 690 samples. Each sample was tested using three different antibody tests: a neutralization assay test and two different ELISA antibody tests. One ELISA test targeted the spike protein and the other targeted the receptor-binding domain (RBD). The neutralization assay test is considered the “gold standard” in antibody testing, so we used these results to identify positive and negative controls [12–14]. This resulted in 245 positive controls and 445 negative controls. Additional details are given elsewhere [15].

The testing dataset consisted of samples from 186 skilled nursing staff during May 2020. Researchers collected one sample from each staff member and ran multiple antibody tests, including the spike and RBD ELISA tests used in the training dataset, as described elsewhere [16].

For both datasets, we normalized the results of the ELISA tests to account for batch effects. Each sample was run twice. We calculated the positive to negative ratio (P/N) by dividing the average optical density for each sample (P) by the average of the negative controls run on the same plate (N) as the sample

1 $$\begin{aligned} \text {P/N ratio} = \frac{\text {Average of samples}}{\text {average of negative samples on plate}}. \end{aligned}$$

This has been described in more detail elsewhere [7, 16].

### Statistical methods to estimate cutoff values

Our objective in determining the cutoff value is to estimate the *Q* quantile of the negative controls for a target specificity of *Q*. Let $\varvec{x}$ denote the vector of *n* negative control test results.

*Normal method*. The normal method finds the *Q* quantile of a normal distribution with a mean of $\bar{x}$ and a standard deviation of $s_x$, where $\bar{x}$ and $s_x$ denote the mean and standard deviation of $\varvec{x}$, respectively [3, 5–7, 17, 18].

*Lognormal method*. The lognormal method is the normal method applied to the data after a natural log transformation [5, 7, 17]. This equates to fitting a lognormal distribution to the raw data and using the *Q* quantile of that lognormal distribution.

*MAD method*. The MAD method is a modification of the normal method that replaces the mean with the median, $\tilde{x}$, and the standard deviation with the scaled mean absolute deviation (MAD), $s_x^{\text {MAD}} = 1.4826 \times \text {median}\{|x_i-\text {median}(\varvec{x})|\}_{i=1}^n$ [5, 6, 18]. This approach is intended to be robust to outliers.

*Log MAD method*. The log MAD method is the MAD method applied to natural log-transformed data [5, 6].

*Empirical method*. The empirical method uses the empirical quantile of $\varvec{x}$ as an estimator of the cutoff, avoiding parametric assumptions [5–7, 17, 18]. The empirical method is the only nonparametric method widely used in the literature and the only one considered herein.

To calculate the *Q* empirical quantile from a sample of size *n*, we calculate a weighted average of the two order statistics surrounding the desired quantile. Specifically, $(1-\gamma )x_{(i)} + \gamma x_{(i + 1)}$ where $x_{(i)}$ denotes the $i^{\text {th}}$ order statistic, $i=\lfloor (n-1) Q + 1\rfloor$, $\gamma =(n-1)Q + 1-\lfloor (n-1)Q + 1\rfloor$, and $\lfloor \cdot \rfloor$ is the floor function.

*Pareto method using the upper 10% (Pareto 0.9) and upper 5% (Pareto 0.95)*. The Pareto method, based on extreme value theory, fits a generalized Pareto distribution to the upper tail of $\varvec{x}$. Like the normal and lognormal methods, this method fits a parametric distribution to the training data. However, it differs from those methods as the Pareto approach fits a parametric distribution only to the upper tail of the distribution of observed data. Hence, the Pareto methods focus on the part of the distribution that we are interested in rather than fitting a distribution to the center of the data and extrapolating to the tails. This approach has been shown to better approximate tail behavior in a variety of settings.

Let *u* denote some threshold, and $\varvec{y}$ be the values in $\varvec{x}$ that exceed *u*. Asymptotically, under regularizing conditions, $\varvec{y}$ follows the generalized Pareto distribution as *u* approaches the upper limit of the distribution for $\varvec{x}$ [8, 19]. The generalized Pareto distribution is

2 $$\begin{aligned} G(y; \sigma _u, \xi ) = \left\{ \begin{array}{ll} 1- \left( 1+\frac{\xi (y-u)}{\sigma _u} \right) ^{-\frac{1}{\xi }} &{} \xi \ne 0 \\ 1- \exp \left( - \frac{y-u}{\sigma _u} \right) &{} \xi =0. \end{array}\right. \end{aligned}$$

We make the simplifying assumption that $\xi =0$, which results in a shifted exponential distribution and has been shown to be preferable for small sample sizes [20]. Thus, we only estimate $\sigma _u$ from the data as *u* is pre-specified.

Following prior literature, we set *u* to be the $k^{\text {th}}$ quantile of $\varvec{x}$ and consider two values of *k*: 90 and 95 [21, 22]. We then fit an exponential distribution to $\varvec{y}-u$. We use maximum likelihood to estimate $\varvec{y}-u \sim \exp (\lambda )$ such that $\hat{\lambda } = \frac{1}{\bar{y}-u}$ where $\bar{y}$ is the sample mean of $\varvec{y}$.

Since $\varvec{y}$ is assumed to be the upper $(100-k)$% of the data, the upper $Q'=\frac{Q-k/100}{1-k/100}$ quantile of our fitted exponential distribution corresponds to the upper *Q* quantile of the data overall. Thus, we set the cutoff as

3 $$C= F^{-1}(Q', \hat{\lambda})+u$$

4 $$= -\hat{\lambda} \log (1-Q') + u,$$

where $F^{-1}(Q', \hat{\lambda })$ is the inverse CDF of an exponential distribution with a scale parameter of $\hat{\lambda }$, evaluated at $Q'$. When $Q=0.95$ and $k=95$, the cutoff estimate is equivalent to the empirical method estimate because $Q'=0$. The threshold *k* should be selected to be sufficiently below *Q*, so $Q'$ itself is not an extreme quantile. The threshold must also be sufficiently large to focus on the upper tail of the distribution.

*Hybrid approaches.* We also consider hybrid approaches that provide a data-driven approach to select a cutoff estimation method [5, 7]. We first test for normality using the Shapiro-Wilk test with a significance level of 0.05. If the test fails to reject, we use the normal method. If the test rejects normality, we natural log transform and test for normality again. If the test fails to reject, we use the lognormal method. If the test rejects normality, we use one of three methods: empirical, Pareto 90%, and Pareto 95% (henceforth referred to as hybrid empirical, hybrid Pareto 0.9, and hybrid Pareto 0.95, respectively).

Additional details on the estimation methods are given in Web Appendix 1.

### Statistical methods to estimate prevalence

To accurately estimate the proportion of the population with antibodies for the disease, the seroprevalence, we account for the sensitivity and specificity of the test via the Rogan-Gladen adjustment [23], modified to disallow any negative estimates. The prevalence estimator is

5 $$\begin{aligned} \hat{\pi } = \text {max}\left( \frac{\hat{p} + Q-1}{Q+\hat{\text {sens}}-1}, 0 \right) , \end{aligned}$$

where $\hat{p}$ is the proportion of tests classified as positive in the testing data and $\hat{\text {sens}}$ denotes the estimated sensitivity of the test: the proportion of the positive controls that correctly tested positive in the training data. We use the target specificity *Q* as the specificity estimate. While it is possible to estimate the specificity, doing so would require splitting the limited training dataset in two, one portion to estimate the cutoff and the second to estimate specificity. Further splitting limited training data is undesirable.

### Data analysis

We used the training dataset to set cutoffs and evaluate the sensitivity. We established cutoffs for both the spike and RBD ELISA tests using two different target specificities: 0.95 and 0.995. For each target specificity and test, we estimated the cutoff using each of the seven methods described above and the three hybrid methods. We used the proportion of training dataset samples with positive neutralization assay results above the cutoff to estimate the sensitivity and the proportion of samples with a negative result below the cutoff to calculate the empirical specificity.

We then used the cutoffs to classify each observation in the testing dataset as positive or negative. The resulting positivity was used to calculate the Rogan-Gladen adjusted prevalence for each cutoff.

### Simulation study

We modeled our simulated data after the training dataset. For each test (spike or RBD) and control type (positive or negative), we fit mixture distributions of the form

6 $$g(x) = \sum\limits_{i=1}^K \pi _i f_i(x)$$

where *K* is the number of components, $\pi _i$ gives the weight of each component, $f_i(x)$ is the probability density function of each component evaluated at *x*, and *g*(*x*) is the resulting mixture distribution evaluated at *x*. We considered gamma, Weibull, and lognormal distributions and either two or three components. All possible combinations of these distribution were fit using the ltmix package in R for each number of components [24]. We selected the best model for each in terms of BIC and visual inspection. The resulting mixture distributions are given in Supplementary Table S1.

We sampled from the fitted mixture distributions to generate data for the simulation study. By sampling from known mixture distributions, we were able to calculate the true quantiles for the population we sampled from, allowing us to assess bias and the root mean squared error (RMSE) of the cutoff value.

We considered eight scenarios in our simulation study. The data was either simulated from the fitted spike P/N ratios distribution (scenario A) or the fitted RBD P/N ratios distribution (scenario B). We varied the training sample size between 50 and 200 controls of each type (positive and negative), resulting in total sample sizes of 100 and 400 and a prevalence of 0.5. We set the target specificity at 0.95 or 0.995. For each simulated training dataset, we generated a corresponding testing dataset of size 500, with the number of positive and negative controls determined by the prevalence: either 0.05 or 0.3. We generated 10,000 training datasets and testing datasets.

For each training dataset, we estimated the cutoff using all seven methods and the three hybrid methods. Then, we estimated the sensitivity of the cutoff using the proportion of the positive controls in the training dataset that were correctly predicted as positive using that cutoff. We also used each cutoff to classify positive and negative results in the testing dataset. We calculated the Rogan-Gladen adjusted prevalence as previously described.

We evaluated the cutoffs in terms of the bias and RMSE. Let $X_{Q,s}$ be the true *Q* percentile of the mixture distribution from which we simulated the negative controls, i.e., the true cutoff with a specificity of *Q*. We calculated the bias for each setting, *s*, as

7 $$\text{Cutoff Bias}_{m,Q,s} = \frac{\sum\nolimits_{i=1}^{10,000} C_{i,m,Q,s}}{10,000} -X_{Q,s},$$

where $C_{i,m,Q,s}$ is the cutoff from the $i^{\text {th}}$ simulated training dataset under setting *s*, using method *m* with a target specificity of *Q*. The RMSE was calculated as

8 $$\text{RMSE}_{m,Q,s} = \sqrt{\frac{\sum\nolimits_{i=1}^{10,000} \left( C_{i,m,Q,s} -X_{Q,s}\right) ^2}{10,000}}.$$

To evaluate the impact of the cutoff method on the inference drawn from the tests (the individual test results and community prevalence estimates), we used accuracy and the bias of the prevalence estimates. We calculated the accuracy of the predictions for the simulated testing datasets as the proportion of testing dataset observations that were correctly predicted for the cutoff of interest. We averaged the accuracies across the 10,000 datasets to estimate the average accuracy for each method, setting, and target quantile combination. The prevalence for each dataset, $\hat{\pi }_{i,m,Q,s}$, was calculated as in (5). For true prevalence $\pi _s$, the bias was calculated as

9 $$\text{Prevalence Bias}_{m,Q,s} = \frac{\sum\nolimits_{i=1}^{10,000} \hat{\pi }_{i,m,Q,s}}{10,000} -\pi _s.$$

## Results

### Data analysis

**Fig. 1:**
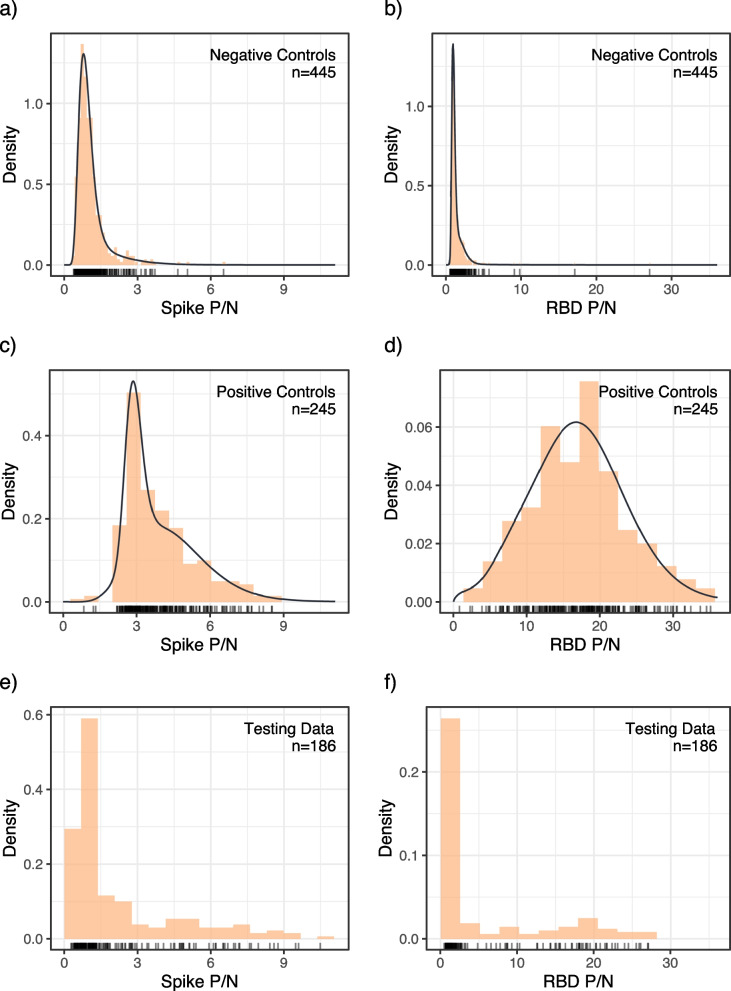
**a**-**d** Histogram of the training dataset for each test and control type overlaid with the corresponding mixture distribution from which the data was generated in the simulation study (training data only). The testing data set are in panels (**e**) and (**f**). The first column corresponds to the spike test, and the second to the receptor-binding domain (RBD) test. Training data was sampled from staff at long-term care facilities in Colorado, USA between June and December 2020. Testing data collected from skilled nursing staff in Colorado during May 2020

Figure 1 shows the negative control training data, positive control training data, and testing data for both the spike and RBD tests. The spike test had a smaller range of P/N ratios and less separation between the positive and negative controls. The RBD negative controls had a sparser upper tail, and the positive controls had a more symmetric distribution compared to the spike test.

#### Spike test

**Fig. 2:**
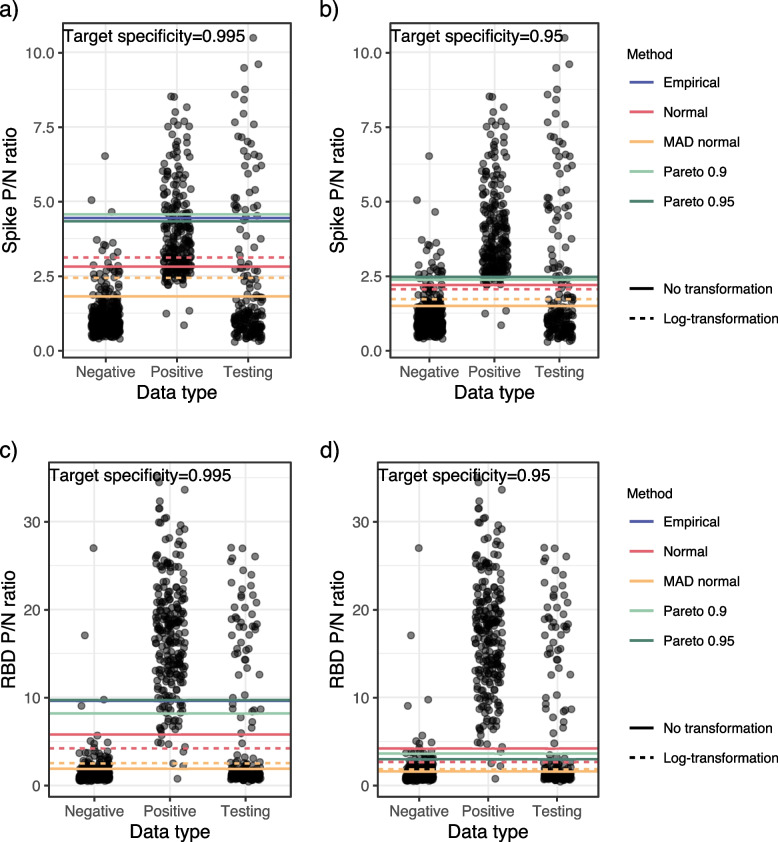
P/N ratios for the positive controls, negative controls, and testing data, jittered horizontally. Cutoffs as calculated by each of the seven methods are shown as horizontal lines. The first row shows the spike test cutoffs with **a** a target specificity of 0.995 and **b** a target specificity of 0.95. The second row shows the receptor-binding domain (RBD) test with **c** a specificity of 0.995 and **d** a target specificity of 0.95. Training data was sampled from staff at long-term care facilities in Colorado, USA between June and December 2020. Testing data collected from skilled nursing staff in Colorado during May 2020

Figure 2 shows the training and testing data and the estimated cutoff for each method, target specificity, and test. Web Appendix 3 shows the results in numerical form. Overall, the different estimation methods resulted in very different cutoff values. When targeting a specificity of 0.995, the spike test cutoffs ranged from 1.8 to 4.6 compared to a range of 1.5 to 2.5 when targeting a specificity of 0.95. The MAD normal methods consistently estimated the lowest cutoffs, while the empirical and Pareto methods resulted in the highest estimates. Using the hybrid approaches, we rejected normality for the untransformed and natural log transformed data and used the empirical and Pareto estimators.

Because the cutoffs are in the tail of the distribution for the negative controls, there are not many negative control observations between the cutoff values from the different methods (Fig. 2). Thus, the differences in the cutoffs have minimal impact on the empirical specificities (Table 1). The cutoffs had a larger impact on the empirical sensitivity because there were many positive controls in the range of the cutoffs as shown in Fig. 2. For example, the Pareto 0.9 and the lognormal cutoffs had similar training data empirical specificities, 0.993 versus 0.978, when targeting a specificity of 0.995. However, the empirical sensitivities were substantially different: 0.27 and 0.63, respectively.

**Table 1:** Rogan-Gladen adjusted prevalence estimate of the testing dataset for each cutoff method, test, and target specificity

	Spike			RBD		
	Prevalence	Sensitivity	Specificity	Prevalence	Sensitivity	Specificity
Target specificity=0.995						
Empirical	0.64	0.28	0.99	0.26	0.87	0.99
Normal	0.30	0.77	0.97	0.28	0.96	0.99
Log Normal	0.35	0.63	0.98	0.28	0.98	0.98
MAD	0.32	0.99	0.91	0.39	1.00	0.85
Log MAD	0.29	0.93	0.95	0.32	0.99	0.93
Pareto 0.9	0.63	0.27	0.99	0.27	0.91	0.99
Pareto 0.95	0.61	0.31	0.99	0.26	0.87	0.99
Target specificity=0.95						
Empirical	0.29	0.93	0.95	0.30	0.99	0.95
Normal	0.30	0.98	0.94	0.28	0.98	0.98
Log Normal	0.31	0.99	0.93	0.31	0.99	0.94
MAD	0.37	0.99	0.85	0.42	1.00	0.80
Log MAD	0.34	0.99	0.90	0.39	1.00	0.84
Pareto 0.9	0.29	0.96	0.94	0.28	0.99	0.97

Empirical and Pareto 0.95 cutoffs are equivalent when the target specificity is 0.95

* Abbreviations*: *MAD* mean absolute deviation, *RBD* receptor-binding domain

The Rogan-Gladen adjusted prevalence estimate for each cutoff method is shown in Table 1. The prevalence estimates from cutoffs targeting a specificity of 0.95 ranged from 0.29 to 0.37. Those targeting 0.995 ranged from 0.29 to 0.64. Most prevalence estimates ranged from 0.26 to 0.42 with either target specificity, but the prevalence estimates from the empirical and Pareto cutoffs targeting a specificity of 0.995 were much larger, between 0.61 and 0.64.

#### RBD test

The estimated cutoffs for the RBD test were also more variable when targeting a specificity of 0.995. The MAD normal cutoffs were the smallest, and the empirical and Pareto cutoffs were similar to each other. We again rejected normality both for the raw and log transformed data, and the hybrid method estimates were equivalent to the empirical and Pareto estimates.

The RBD test showed greater separation in the distributions of the negative controls and positive controls, resulting in higher and more consistent empirical sensitivities, with all sensitivities greater than 0.87 (Table 1). The reduced variability in the empirical sensitivity estimates between estimation methods resulted in less variability of the prevalence estimates, compared to the spike tests.

### Simulation study

Figure 1a-d show the distribution functions we generated data from. There was more overlap between the positive and negative cases in the data for scenario A than in scenario B. This is partially a result of the right skew of the positive controls and partially because the tail of the negative controls extends further in scenario A than in scenario B.

#### Cutoff estimation

Table 2 shows the bias and the RMSE of the cutoff for each method when targeting a specificity of 0.995. In the majority of cases, the Pareto methods were superior in terms of bias and RMSE. The only exception was scenario B with a training sample size of 50 where the RMSE was smallest for the lognormal method because the larger bias for this method was offset by the smaller variance.

**Table 2:** The mean and Monte Carlo standard error in parentheses of the bias and RMSE of the cutoff when targeting a specificity of 0.995. The method(s) with minimal bias and RMSE in each scenario or equivalent after rounding are bolded

	Scenario A		Scenario B	
	n=50	n=200	n=50	n=200
Bias of cutoff				
Empirical	-0.93 (0.0049)	-0.37 (0.0035)	-3.60 (0.0327)	-1.77 (0.0249)
Normal	-1.74 (0.0027)	-1.70 (0.0014)	-6.26 (0.0158)	-5.71 (0.0112)
Log Normal	-1.34 (0.0030)	-1.36 (0.0015)	-6.72 (0.0070)	-6.79 (0.0032)
MAD	-2.68 (0.0010)	-2.68 (0.0005)	-9.08 (0.0013)	-9.09 (0.0006)
Log MAD	-2.00 (0.0025)	-2.02 (0.0012)	-8.41 (0.0031)	-8.47 (0.0014)
Pareto 0.9	**-0.15 (0.0064)**	**-0.02 (0.0033)**	-3.03 (0.0309)	-2.97 (0.0154)
Pareto 0.95	-0.50 (0.0059)	**-0.02 (0.0035)**	**-2.73 (0.0368)**	**-1.35 (0.0227)**
Hybrid Empirical	-0.93 (0.0048)	-0.38 (0.0036)	-3.59 (0.0327)	-1.77 (0.0249)
Hybrid Pareto 0.9	-0.37 (0.0069)	-0.03 (0.0033)	-3.06 (0.0310)	-2.97 (0.0154)
Hybrid Pareto 0.95	-0.62 (0.0061)	-0.03 (0.0035)	-2.74 (0.0368)	**-1.35 (0.0227)**
RMSE of cutoff				
Empirical	1.35 (0.0039)	0.80 (0.0031)	7.47 (0.0789)	5.28 (0.0360)
Normal	1.82 (0.0024)	1.72 (0.0014)	7.01 (0.0180)	6.14 (0.0090)
Log Normal	1.47 (0.0026)	1.39 (0.0014)	**6.86 (0.0059)**	6.82 (0.0031)
MAD	2.69 (0.0010)	2.68 (0.0005)	9.08 (0.0013)	9.09 (0.0006)
Log MAD	2.07 (0.0022)	2.04 (0.0012)	8.43 (0.0030)	8.48 (0.0014)
Pareto 0.9	**1.28 (0.0059)**	**0.65 (0.0029)**	6.88 (0.0619)	**4.28 (0.0133)**
Pareto 0.95	**1.28 (0.0052)**	0.70 (0.0033)	7.84 (0.0871)	4.74 (0.0294)
Hybrid Empirical	1.34 (0.0037)	0.80 (0.0031)	7.45 (0.0790)	5.28 (0.0360)
Hybrid Pareto 0.9	1.43 (0.0057)	0.66 (0.0030)	6.91 (0.0616)	**4.28 (0.0133)**
Hybrid Pareto 0.95	1.37 (0.0050)	0.71 (0.0033)	7.85 (0.0870)	4.74 (0.0294)

*Abbreviations*: *MAD *mean absolute deviation, *RMSE* root mean squared error

The cutoff estimates with every method were negatively biased, meaning the cutoff was below the true 0.995 quantile for each method, on average. Thus, the specificity of the estimated cutoff was below the target, on average. The MAD and log MAD methods were the most biased while the Pareto methods were the least biased.

The hybrid methods all had slightly higher RMSE and bias than their corresponding Pareto or empirical methods. Normality and log normality were both rejected for the vast majority of the datasets: 99-100% of datasets with a training sample size of 200 and 59-92% with a training sample size of 50. The results are, therefore, mostly the Pareto and empirical cutoffs but with a small number of poorer performing normal or lognormal cutoffs mixed in.

Table 3 shows, when targeting a specificity of 0.95, the magnitude of the bias and RMSE were smaller. The empirical method had the minimal bias under scenario B. The Pareto 0.9 and normal methods had a positive bias for scenario B, compared to the negative bias when targeting a specificity of 0.995.

**Table 3:** The mean and Monte Carlo standard error in parentheses of the bias and RMSE of the cutoff when targeting a specificity of 0.95. The method(s) with minimal bias and RMSE in each scenario or equivalent after rounding are bolded

	Scenario A		Scenario B	
	n=50	n=200	n=50	n=200
Bias of cutoff				
Empirical	-0.14 (0.0025)	-0.05 (0.0014)	**-0.02 (0.0041)**	**-0.02 (0.0015)**
Normal	-0.26 (0.0018)	-0.23 (0.0010)	0.65 (0.0105)	1.00 (0.0073)
Log Normal	-0.35 (0.0014)	-0.36 (0.0007)	-0.23 (0.0029)	-0.24 (0.0014)
MAD	-0.92 (0.0007)	-0.92 (0.0004)	-1.29 (0.0009)	-1.29 (0.0004)
Log MAD	-0.68 (0.0012)	-0.68 (0.0006)	-1.03 (0.0015)	-1.06 (0.0007)
Pareto 0.9	**-0.07 (0.0022)**	**-0.03 (0.0012)**	0.65 (0.0077)	0.70 (0.0038)
Hybrid Empirical	-0.17 (0.0024)	-0.05 (0.0014)	-0.03 (0.0041)	**-0.02 (0.0015)**
Hybrid Pareto 0.9	-0.12 (0.0023)	**-0.03 (0.0012)**	0.64 (0.0077)	0.70 (0.0038)
RMSE of cutoff				
Empirical	0.51 (0.0021)	0.29 (0.0012)	0.82 (0.0130)	**0.31 (0.0017)**
Normal	**0.45 (0.0015)**	0.30 (0.0008)	2.20 (0.0341)	1.77 (0.0170)
Log Normal	**0.45 (0.0012)**	0.38 (0.0007)	**0.63 (0.0028)**	0.37 (0.0011)
MAD	0.93 (0.0007)	0.93 (0.0004)	1.30 (0.0009)	1.30 (0.0004)
Log MAD	0.72 (0.0011)	0.69 (0.0006)	1.08 (0.0013)	1.07 (0.0007)
Pareto 0.9	**0.45 (0.0019)**	**0.23 (0.0010)**	1.67 (0.0182)	1.04 (0.0056)
Hybrid Empirical	0.51 (0.0020)	0.29 (0.0012)	0.83 (0.0129)	**0.31 (0.0017)**
Hybrid Pareto 0.9	0.47 (0.0019)	0.24 (0.0010)	1.67 (0.0181)	1.04 (0.0056)

Empirical and Pareto 0.95 cutoffs are equivalent when the target specificity is 0.95

* Abbreviations*: *MAD* mean absolute deviation, *RMSE* root mean squared error

#### Prevalence estimation

Table 4 shows simulation results for the Rogan-Gladen adjusted prevalence estimates when targeting a specificity of 0.995. The Pareto cutoffs had little bias but had larger variability when targeting a specificity of 0.995. In every case, the average of the prevalence point estimates was closest to the truth using one of the Pareto methods. However, in scenario A the Pareto estimates, especially with a sample size of 50, were more variable than the normal-based methods. Table 5 shows the prevalence when targeting a specificity of 0.95. The variability for the Pareto and empirical methods were lower when targeting a lower specificity, and particularly at the smaller sample size. With both target specificities, the MAD and log MAD methods were positively biased, while the other methods had a smaller bias, generally positive. The hybrid method estimates were again similar to the corresponding empirical and Pareto estimates.

**Table 4:** The bias and RMSE in parentheses of the Rogan-Gladen adjusted prevalence estimates when targeting a specificity of 0.995. The method(s) with the smallest bias in each scenario or equivalent after rounding are bolded

	Scenario A				Scenario B			
	n=50		n=200		n=50		n=200	
Prevalence=0.05								
Empirical	0.02	(0.0328)	0.01	(0.0212)	**0.02**	**(0.0315)**	**0.00**	**(0.0119)**
Normal	0.04	(0.0406)	0.04	(0.0362)	0.03	(0.0300)	0.01	(0.0145)
Log Normal	0.03	(0.0317)	0.03	(0.0291)	**0.02**	**(0.0213)**	0.02	(0.0159)
MAD	0.09	(0.0911)	0.09	(0.0878)	0.14	(0.1374)	0.14	(0.1361)
Log MAD	0.05	(0.0499)	0.05	(0.0461)	0.08	(0.0754)	0.07	(0.0695)
Pareto 0.9	**0.01**	**(0.0453)**	**0.00**	**(0.0204)**	**0.02**	**(0.0247)**	**0.00**	**(0.0074)**
Pareto 0.95	0.02	(0.0382)	**0.00**	**(0.0213)**	**0.02**	**(0.0346)**	**0.00**	**(0.0096)**
Hybrid Empirical	0.02	(0.0322)	0.01	(0.0212)	**0.02**	**(0.0311)**	**0.00**	**(0.0119)**
Hybrid Pareto 0.9	0.02	(0.0478)	**0.00**	**(0.0205)**	**0.02**	**(0.0255)**	**0.00**	**(0.0074)**
Hybrid Pareto 0.95	0.02	(0.0395)	**0.00**	**(0.0214)**	0.03	(0.0348)	**0.00**	**(0.0096)**
Prevalence=0.30								
Empirical	0.02	(0.0563)	0.01	(0.0396)	**0.01**	**(0.0309)**	**0.00**	**(0.0161)**
Normal	0.03	(0.0390)	0.03	(0.0295)	0.02	(0.0244)	0.01	(0.0125)
Log Normal	0.03	(0.0418)	0.02	(0.0290)	0.02	(0.0173)	0.01	(0.0122)
MAD	0.07	(0.0676)	0.06	(0.0646)	0.10	(0.1012)	0.10	(0.1003)
Log MAD	0.04	(0.0420)	0.03	(0.0343)	0.06	(0.0553)	0.05	(0.0512)
Pareto 0.9	**0.01**	**(0.0976)**	**0.00**	**(0.0438)**	**0.01**	**(0.0293)**	**0.00**	**(0.0105)**
Pareto 0.95	0.02	(0.0749)	**0.00**	**(0.0447)**	**0.01**	**(0.0349)**	**0.00**	**(0.0154)**
Hybrid Empirical	0.02	(0.0554)	0.01	(0.0396)	**0.01**	**(0.0305)**	**0.00**	**(0.0161)**
Hybrid Pareto 0.9	**0.01**	**(0.0922)**	**0.00**	**(0.0438)**	**0.01**	**(0.0298)**	**0.00**	**(0.0105)**
Hybrid Pareto 0.95	0.02	(0.0722)	**0.00**	**(0.0447)**	**0.01**	**(0.0350)**	**0.00**	**(0.0154)**

*Abbreviations*: *MAD* mean absolute deviation

**Table 5:** The bias and RMSE in parentheses of the Rogan-Gladen adjusted prevalence estimates when targeting a specificity of 0.95. The method(s) with the smallest bias in each scenario or equivalent after rounding are bolded

	Scenario A				Scenario B			
	n=50		n=200		n=50		n=200	
Prevalence=0.05								
Empirical	**0.01**	**(0.0323)**	**0.00**	**(0.0173)**	0.02	(0.0293)	**0.00**	**(0.0152)**
Normal	0.02	(0.0287)	0.02	(0.0186)	0.01	(0.0404)	-0.01	(0.0260)
Log Normal	0.03	(0.0292)	0.02	(0.0242)	0.03	(0.0369)	0.02	(0.0236)
MAD	0.10	(0.1049)	0.10	(0.0997)	0.16	(0.1558)	0.15	(0.1536)
Log MAD	0.06	(0.0621)	0.06	(0.0566)	0.11	(0.1129)	0.11	(0.1114)
Pareto 0.9	**0.01**	**(0.0263)**	**0.00**	**(0.0146)**	**0.00**	**(0.0298)**	-0.02	(0.0215)
Hybrid Empirical	**0.01**	**(0.0324)**	**0.00**	**(0.0173)**	0.02	(0.0301)	**0.00**	**(0.0152)**
Hybrid Pareto 0.9	**0.01**	**(0.0286)**	**0.00**	**(0.0147)**	**0.00**	**(0.0308)**	-0.02	(0.0215)
Prevalence=0.30								
Empirical	**0.01**	**(0.0281)**	**0.00**	**(0.0153)**	0.01	(0.0223)	**0.00**	**(0.0122)**
Normal	0.02	(0.0245)	0.01	(0.0153)	0.01	(0.0295)	-0.01	(0.0196)
Log Normal	0.02	(0.0239)	0.02	(0.0186)	0.02	(0.0280)	0.01	(0.0179)
MAD	0.08	(0.0781)	0.07	(0.0732)	0.11	(0.1146)	0.11	(0.1133)
Log MAD	0.05	(0.0468)	0.04	(0.0415)	0.08	(0.0830)	0.08	(0.0822)
Pareto 0.9	**0.01**	**(0.0244)**	**0.00**	**(0.0135)**	**0.00**	**(0.0225)**	-0.01	(0.0164)
Hybrid Empirical	**0.01**	**(0.0279)**	**0.00**	**(0.0153)**	0.01	(0.0229)	**0.00**	**(0.0122)**
Hybrid Pareto 0.9	**0.01**	**(0.0256)**	**0.00**	**(0.0135)**	**0.00**	**(0.0233)**	-0.01	(0.0164)

Empirical and Pareto 0.95 cutoffs are equivalent when the target specificity is 0.95

* Abbreviations*: *MAD* mean absolute deviation

#### Test accuracy

We consider the accuracy of the cutoff estimation methods for classifying individuals as positive or negative in the testing data. Tables 6 and 7 show the proportion of testing set observations correctly classified with a target specificity of 0.995 and 0.95, respectively. The MAD methods’ cutoffs were negatively biased, leading to a lower specificity and decreased accuracy in low prevalence scenarios. The Pareto methods had the highest accuracy (or equivalent to the highest accuracy) when prevalence was 0.05.

**Table 6:** The mean and middle 95% (2.5% quantile, 97.5% quantile) of the accuracy of the test as measured by the proportion of testing dataset observations correctly predicted when targeting a specificity of 0.995. The method(s) with highest accuracy in each scenario or equivalent after rounding are bolded

	Scenario A				Scenario B			
	n=50		n=200		n=50		n=200	
Prevalence=0.05								
Empirical	**0.96**	**(0.93, 0.97)**	**0.96**	**(0.95, 0.97)**	0.97	(0.93, 0.99)	0.98	(0.96, 0.99)
Normal	0.95	(0.91, 0.97)	**0.96**	**(0.93, 0.97)**	0.97	(0.90, 0.99)	0.98	(0.95, 0.99)
Log Normal	**0.96**	**(0.93, 0.97)**	**0.96**	**(0.94, 0.97)**	0.97	(0.93, 0.99)	0.98	(0.96, 0.99)
MAD	0.90	(0.84, 0.95)	0.91	(0.87, 0.94)	0.86	(0.78, 0.93)	0.86	(0.81, 0.90)
Log MAD	0.94	(0.89, 0.97)	0.95	(0.92, 0.97)	0.92	(0.83, 0.98)	0.93	(0.87, 0.97)
Pareto 0.9	**0.96**	**(0.94, 0.97)**	**0.96**	**(0.95, 0.97)**	**0.98**	**(0.95, 0.99)**	**0.99**	**(0.97, 1.00)**
Pareto 0.95	**0.96**	**(0.93, 0.97)**	**0.96**	**(0.95, 0.97)**	**0.98**	**(0.94, 0.99)**	0.98	(0.96, 0.99)
Hybrid Empirical	**0.96**	**(0.93, 0.97)**	**0.96**	**(0.95, 0.97)**	0.97	(0.93, 0.99)	0.98	(0.96, 0.99)
Hybrid Pareto 0.9	**0.96**	**(0.93, 0.97)**	**0.96**	**(0.95, 0.97)**	**0.98**	**(0.94, 0.99)**	**0.99**	**(0.97, 1.00)**
Hybrid Pareto 0.95	**0.96**	**(0.93, 0.97)**	**0.96**	**(0.95, 0.97)**	**0.98**	**(0.94, 0.99)**	0.98	(0.96, 0.99)
Prevalence=0.30								
Empirical	0.85	(0.73, 0.95)	0.81	(0.73, 0.89)	0.95	(0.74, 0.99)	0.94	(0.77, 0.99)
Normal	0.91	(0.81, 0.96)	0.91	(0.84, 0.96)	0.96	(0.90, 0.99)	0.97	(0.94, 0.99)
Log Normal	0.88	(0.78, 0.96)	0.87	(0.81, 0.94)	**0.97**	**(0.94, 0.99)**	**0.98**	**(0.96, 0.99)**
MAD	**0.93**	**(0.88, 0.96)**	0.93	(0.90, 0.95)	0.89	(0.84, 0.95)	0.90	(0.86, 0.93)
Log MAD	0.92	(0.82, 0.96)	**0.94**	**(0.88, 0.96)**	0.94	(0.87, 0.98)	0.94	(0.90, 0.98)
Pareto 0.9	0.81	(0.71, 0.95)	0.79	(0.73, 0.86)	0.95	(0.74, 0.99)	0.96	(0.87, 0.99)
Pareto 0.95	0.82	(0.71, 0.95)	0.79	(0.73, 0.86)	0.94	(0.72, 0.99)	0.94	(0.78, 0.99)
Hybrid Empirical	0.85	(0.73, 0.95)	0.81	(0.73, 0.89)	0.95	(0.74, 0.99)	0.94	(0.77, 0.99)
Hybrid Pareto 0.9	0.82	(0.71, 0.95)	0.79	(0.73, 0.86)	0.95	(0.74, 0.99)	0.96	(0.87, 0.99)
Hybrid Pareto 0.95	0.83	(0.71, 0.95)	0.79	(0.73, 0.86)	0.94	(0.72, 0.99)	0.94	(0.78, 0.99)

*Abbreviations*: *MAD* mean absolute deviation

**Table 7:** The mean and middle 95% (2.5% quantile, 97.5% quantile) of the accuracy of the test as measured by the proportion of testing dataset observations correctly predicted when targeting a specificity of 0.95. The method(s) with highest accuracy in each scenario or equivalent after rounding are bolded

	Scenario A				Scenario B			
	n=50		n=200		n=50		n=200	
Prevalence=0.05								
Empirical	0.93	(0.86, 0.97)	0.94	(0.91, 0.97)	0.94	(0.86, 0.99)	0.95	(0.91, 0.98)
Normal	0.93	(0.87, 0.97)	0.94	(0.90, 0.96)	0.94	(0.85, 0.99)	0.96	(0.90, 0.99)
Log Normal	0.93	(0.87, 0.96)	0.93	(0.90, 0.96)	0.93	(0.84, 0.98)	0.93	(0.89, 0.97)
MAD	0.85	(0.76, 0.92)	0.86	(0.81, 0.90)	0.80	(0.72, 0.88)	0.81	(0.76, 0.85)
Log MAD	0.89	(0.81, 0.95)	0.90	(0.86, 0.94)	0.85	(0.75, 0.94)	0.85	(0.79, 0.90)
Pareto 0.9	**0.94**	**(0.88, 0.97)**	**0.95**	**(0.91, 0.97)**	**0.95**	**(0.87, 0.99)**	**0.97**	**(0.93, 0.99)**
Hybrid Empirical	0.93	(0.86, 0.97)	0.94	(0.91, 0.97)	0.94	(0.85, 0.99)	0.95	(0.91, 0.98)
Hybrid Pareto 0.9	**0.94**	**(0.88, 0.97)**	**0.95**	**(0.91, 0.97)**	**0.95**	**(0.86, 0.99)**	**0.97**	**(0.93, 0.99)**
Prevalence=0.30								
Empirical	0.93	(0.84, 0.96)	**0.94**	**(0.88, 0.96)**	0.95	(0.89, 0.99)	0.96	(0.93, 0.98)
Normal	0.93	(0.88, 0.96)	**0.94**	**(0.92, 0.96)**	0.95	(0.88, 0.99)	**0.97**	**(0.92, 0.99)**
Log Normal	**0.94**	**(0.90, 0.96)**	**0.94**	**(0.92, 0.96)**	0.94	(0.88, 0.98)	0.95	(0.91, 0.98)
MAD	0.89	(0.82, 0.94)	0.89	(0.86, 0.93)	0.86	(0.79, 0.91)	0.86	(0.82, 0.89)
Log MAD	0.92	(0.86, 0.96)	0.92	(0.89, 0.95)	0.89	(0.82, 0.95)	0.89	(0.84, 0.93)
Pareto 0.9	0.93	(0.85, 0.96)	**0.94**	**(0.90, 0.96)**	**0.96**	**(0.90, 0.99)**	**0.97**	**(0.94, 0.99)**
Hybrid Empirical	0.93	(0.84, 0.96)	**0.94**	**(0.88, 0.96)**	0.95	(0.89, 0.99)	0.96	(0.93, 0.98)
Hybrid Pareto 0.9	0.93	(0.85, 0.96)	**0.94**	**(0.90, 0.96)**	**0.96**	**(0.90, 0.99)**	**0.97**	**(0.94, 0.99)**

Empirical and Pareto 0.95 cutoffs are equivalent when the target specificity is 0.95

* Abbreviations*: *MAD* mean absolute deviation

When the prevalence was higher at 0.3 and using the lower target specificity, the Pareto method was most accurate in scenario B. All but the MAD methods performed similarly for scenario A. With the higher target specificity, the MAD cutoffs had highest accuracy for scenario A, and the lognormal method was most accurate for scenario B.

## Discussion

It is imperative to rapidly develop and deploy prognostic tests for emerging infectious diseases that can be used to classify individuals and estimate prevalence in a community. A common challenge for tests is determining a cutoff value to separate positive and negative cases as there is often overlap in the results between the positive and negative cases. This is especially challenging with early tests for emerging diseases for which there is limited training data with validated positive and negative controls. Common approaches to estimating cutoff values are using the quantile of a parametric distribution fit to the negative control test data or using the empirical quantile of the negative control test data. Yet, there is little guidance on how to select a cutoff to separate positive and negative results, especially for small data sets. Here, we proposed using methods from extreme value theory, specifically using the generalized Pareto distribution to estimate the upper tail of the negative control training data and its quantiles, to estimate a cutoff value to achieve a target specificity. We compared the proposed approach and common alternatives in a simulation study.

Our simulation demonstrated that when targeting a very high specificity, 0.995 as recommended by the CDC early in the COVID-19 pandemic [4], the Pareto methods proposed had lower bias and RMSE for estimating a cutoff value. When targeting a lower target specificity of 0.95, the empirical method consistently performed well. Methods that relied on parametric distributions (e.g., normal, lognormal, MAD normal and MAD lognormal) generally had large bias and RMSE.

Additionally, we compared the recommended target specificity of 0.995 to a target specificity of 0.95 and found the desired target specificity varied according to the goal of the analysis as well as the prevalence of the population. In the low prevalence setting we might expect for an emerging disease, using a higher target cutoff of 0.995, as compared to the more moderate 0.95, resulted in better accuracy for classifying individuals as positive or negative (Tables 6 and 7). With higher prevalence, accuracy was overall higher when targeting a specificity of 0.95 instead of 0.995. We also found the variability of the prevalence estimate was generally lower for the empirical and Pareto methods when targeting a specificity of 0.95.

The results of our data analysis of two COVID-19 antibody tests are consistent with the results of the simulation study. The Pareto and empirical methods, which showed minimal negative bias in the simulation study, also tended to have the highest cutoff estimates in the data analysis. The MAD methods showed considerable negative bias in the simulation study and had the smallest estimates in the data analysis. Additionally, like the simulation study, the prevalence estimates showed more variability when targeting a specificity of 0.995 rather than 0.95.

The performance of the cutoff estimators and the resulting accuracy at the individual level and prevalence estimators at the community-levels will vary depending on the shape of the distributions of positive and negative results and the separation between those two distributions. The shape of the distribution impacts how accurately the target specificity can be estimated for the methods using parametric assumptions. The separation of the distributions impacts accuracy, sensitivity, and prevalence estimates. If the distributions show considerable overlap, the accuracy is lowered, and a cutoff cannot be selected that results in both a highly sensitive and highly specific test. We only generated data from two possible distributions and two possible sample sizes, so the results of our simulation study should be limited to this context. Considering the data analysis, the neutralization assay test we used to classify positive and negative controls is itself imperfect. The training dataset classifications in our data analysis could be incorrect, which would impact the cutoff estimates.

Because an emerging disease has potential cross-reactivity and few true positives expected, we focus on methods for establishing cutoffs that target a high specificity [2]. However, in other applications, approaches that consider both the sensitivity and specificity, as well as the relative costs of false positive and false negative results and the prevalence, may be preferred [25–28]. There are also hypothesis testing-based approaches found in the optimal cutoff selection for patient segmentation literature focused on maximizing statistical power between the groups formed by the cutoff, while controlling the Type I error rate [29–31]. This is a distinct problem from estimating high specificity cutoffs from a sample of validated negative controls, and as such, the methods presented here are not appropriate for this problem. When only estimating prevalence, some methods forgo establishing a cutoff and instead fit a mixture model [32–36] or a latent class model [37–39] to the continuous test results. In some situations, training data may be continuously collected. Users may consider streaming algorithms for quantile estimation in these situations [40, 41].

Based on our simulation and data analysis, we recommend using the Pareto methods or the empirical method to estimate the cutoff when developing tests, depending on the target specificity. The commonly used normal and MAD normal methods showed a larger bias in our simulations. The choice of target specificity of the cutoff should account for the goals of the test. Higher target specificity is preferred when prevalence is very low and the objective is to identify cases, and lower target specificity is preferred if the goal is estimating prevalence.

### Supplementary Information

**Additional file 1.** See the online Supplementary Materials for technical appendices and additional results.

## Data Availability

The datasets analysed during the current study are available in the GitHub repository, https://github.com/pughs/cutoff-selection.

## Abbreviations

CDC: Centers for Disease Control and Prevention
COVID-19: Coronavirus disease 2019
RBD: Receptor-binding domain
ELISA: Enzyme linked immunosorbent assay
MAD: Mean absolute deviation
RMSE: Root mean squared error
SARS-CoV-2: Severe acute respiratory syndrome coronavirus 2
