# Supplementary material for: Estimating cutoff values for diagnostic tests to achieve target specificity using extreme value theory
Source: BMC Med Res Methodol. 2024 Feb 8;24:30. doi: 10.1186/s12874-023-02139-5 (PMC10851584; doi:10.1186/s12874-023-02139-5)
Supplement: Supplementary file 1 — Additional file 1. See the online Supplementary Materials for technical appendices and additional results. [file 12874_2023_2139_MOESM1_ESM.pdf]

Supplemental Material for *Estimating cutoff values for diagnostic tests to achieve target specificity using extreme value theory*

1. CUTOFF ESTIMATION METHODS

1.1. Hybrid methods

Figure S1 shows the flow chart for the hybrid methods.

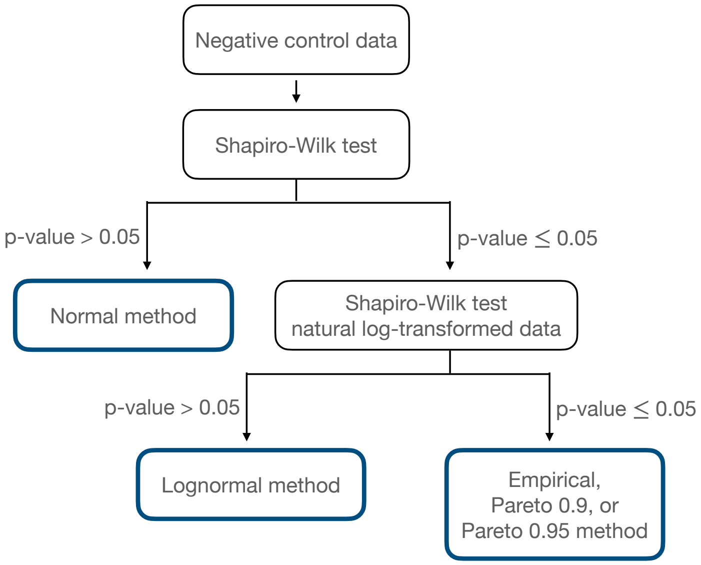

Figure S1. Flow chart indicating how the cutoff is estimated for the hybrid methods.

2. MIXTURE DISTRIBUTION FOR THE SIMULATION STUDY

Table S1. The mixture distribution fit to each test and control type. The mixture probabilities are given by π_i.

Test	Control type	π_1	Distribution 1	π_2	Distribution 2	π_3	Distribution 3
Spike	Positive	0.65	gamma(8.42, 0.53)	0.35	lognormal(1.06, 0.12)		
	Negative	0.13	lognormal(0.75, 0.42)	0.87	lognormal(-0.13, 0.32)		
RBD	Positive	0.36	gamma(5.87, 2.32)	0.59	gamma(13.33, 1.48)	0.05	gamma(1.67, 4.71)
	Negative	0.27	gamma(6.62, 0.29)	0.69	lognormal(-0.05, 0.22)	0.03	lognormal(1.49, 0.88)

Abbreviations: receptor-binding domain, RBD

3. DATA ANALYSIS CUTOFFS

Table S2 gives the results from Figure 2 in the main text in numerical form. Specifically, it gives the cutoffs for each test, method, and target specificity.

Table S2. Estimated cutoff for each estimation method on each training data source.

	Empirical	Normal	Log Normal	MAD	Log MAD	Pareto 0.9	Pareto 0.95
Target specificity=0.95							
Spike	2.5	2.2	2.1	1.5	1.7	2.4	2.5
RBD	3.0	4.2	2.7	1.6	1.8	3.6	3.0
Target specificity=0.995							
Spike	4.4	2.8	3.1	1.8	2.4	4.6	4.3
RBD	9.6	5.8	4.2	1.9	2.5	8.2	9.7

Abbreviations: mean absolute deviation, MAD; receptor-binding domain, RBD

4. SENSITIVITY ESTIMATES

Table S3. The median and middle 95% (2.5% quantile, 97.5% quantile) of the sensitivity. The method with the largest sensitivity in each scenario is bolded.

	Scenario A				Scenario B			
	n=50		n=200		n=50		n=200	
Target specificity=0.995								
Empirical	0.51	(0.11, 0.98)	0.38	(0.13, 0.69)	0.97	(0.12, 0.99)	0.91	(0.23, 0.99)
Normal	0.84	(0.39, 0.99)	0.80	(0.52, 0.96)	0.98	(0.71, 0.99)	0.97	(0.81, 0.99)
Log Normal	0.64	(0.30, 0.97)	0.62	(0.43, 0.88)	0.98	(0.92, 0.99)	0.98	(0.96, 0.99)
MAD	**0.99**	**(0.96, 1.00)**	**0.99**	**(0.98, 0.99)**	**0.99**	**(0.99, 1.00)**	**0.99**	**(0.99, 1.00)**
Log MAD	0.94	(0.44, 0.99)	0.93	(0.69, 0.98)	0.99	(0.98, 1.00)	0.99	(0.99, 0.99)
Pareto 0.9	0.35	(0.03, 0.96)	0.31	(0.12, 0.55)	0.96	(0.12, 0.99)	0.93	(0.56, 0.98)
Pareto 0.95	0.42	(0.05, 0.97)	0.31	(0.10, 0.57)	0.96	(0.05, 0.99)	0.90	(0.25, 0.98)
Hybrid Empirical	0.52	(0.11, 0.97)	0.38	(0.13, 0.70)	0.97	(0.12, 0.99)	0.91	(0.23, 0.99)
Hybrid Pareto 0.9	0.40	(0.03, 0.97)	0.31	(0.12, 0.56)	0.96	(0.12, 0.99)	0.93	(0.56, 0.98)
Hybrid Pareto 0.95	0.45	(0.05, 0.97)	0.31	(0.10, 0.58)	0.96	(0.05, 0.99)	0.90	(0.25, 0.98)
Target specificity=0.95								
Empirical	0.97	(0.52, 1.00)	0.95	(0.69, 0.99)	0.99	(0.97, 0.99)	0.99	(0.99, 0.99)
Normal	0.98	(0.69, 1.00)	0.97	(0.88, 0.99)	0.99	(0.88, 0.99)	0.99	(0.92, 0.99)
Log Normal	0.98	(0.83, 1.00)	0.98	(0.95, 0.99)	0.99	(0.98, 0.99)	0.99	(0.99, 0.99)
MAD	**1.00**	**(0.99, 1.00)**	**1.00**	**(0.99, 1.00)**	**1.00**	**(0.99, 1.00)**	**1.00**	**(1.00, 1.00)**
Log MAD	0.99	(0.96, 1.00)	0.99	(0.98, 1.00)	0.99	(0.99, 1.00)	0.99	(0.99, 1.00)
Pareto 0.9	0.96	(0.54, 1.00)	0.94	(0.74, 0.99)	0.99	(0.92, 0.99)	0.99	(0.97, 0.99)
Hybrid Empirical	0.97	(0.52, 1.00)	0.95	(0.69, 0.99)	0.99	(0.97, 0.99)	0.99	(0.99, 0.99)
Hybrid Pareto 0.9	0.97	(0.54, 1.00)	0.94	(0.74, 0.99)	0.99	(0.92, 0.99)	0.99	(0.97, 0.99)

Abbreviations: mean absolute deviation, MAD

5. PREVALENCE ESTIMATES

Table S4. The mean and middle 95% (2.5% quantile, 97.5% quantile) of the Rogan-Gladen adjusted prevalence estimates when targeting a specificity of 0.995. The method(s) with the prevalence estimate nearest the truth in each scenario or equivalent after rounding are bolded.

	Scenario A				Scenario B			
	n=50		n=200		n=50		n=200	
**Prevalence=0.05**								
Empirical	0.07	(0.01, 0.13)	0.06	(0.00, 0.10)	**0.07**	**(0.02, 0.13)**	**0.05**	**(0.03, 0.08)**
Normal	0.09	(0.05, 0.13)	0.09	(0.06, 0.11)	0.08	(0.04, 0.15)	0.06	(0.05, 0.10)
Log Normal	0.08	(0.04, 0.12)	0.08	(0.05, 0.11)	**0.07**	**(0.05, 0.12)**	0.07	(0.05, 0.09)
MAD	0.14	(0.10, 0.20)	0.14	(0.11, 0.17)	0.19	(0.11, 0.26)	0.19	(0.14, 0.23)
Log MAD	0.10	(0.06, 0.15)	0.10	(0.07, 0.13)	0.13	(0.06, 0.22)	0.12	(0.08, 0.17)
Pareto 0.9	**0.06**	**(0.00, 0.14)**	**0.05**	**(0.00, 0.10)**	**0.07**	**(0.02, 0.10)**	**0.05**	**(0.04, 0.07)**
Pareto 0.95	0.07	(0.00, 0.13)	**0.05**	**(0.00, 0.10)**	**0.07**	**(0.01, 0.13)**	**0.05**	**(0.03, 0.07)**
Hybrid Empirical	0.07	(0.01, 0.12)	0.06	(0.00, 0.10)	**0.07**	**(0.02, 0.13)**	**0.05**	**(0.03, 0.08)**
Hybrid Pareto 0.9	0.07	(0.00, 0.14)	**0.05**	**(0.00, 0.10)**	**0.07**	**(0.02, 0.11)**	**0.05**	**(0.04, 0.07)**
Hybrid Pareto 0.95	0.07	(0.00, 0.13)	**0.05**	**(0.00, 0.10)**	0.08	(0.01, 0.13)	**0.05**	**(0.03, 0.07)**
**Prevalence=0.30**								
Empirical	0.32	(0.19, 0.48)	0.31	(0.21, 0.41)	**0.31**	**(0.24, 0.38)**	**0.30**	**(0.26, 0.34)**
Normal	0.33	(0.25, 0.41)	0.33	(0.28, 0.37)	0.32	(0.28, 0.37)	0.31	(0.29, 0.34)
Log Normal	0.33	(0.23, 0.43)	0.32	(0.26, 0.38)	0.32	(0.29, 0.35)	0.31	(0.30, 0.33)
MAD	0.37	(0.33, 0.42)	0.36	(0.34, 0.39)	0.40	(0.34, 0.46)	0.40	(0.36, 0.44)
Log MAD	0.34	(0.27, 0.40)	0.33	(0.30, 0.36)	0.36	(0.30, 0.43)	0.35	(0.32, 0.39)
Pareto 0.9	**0.31**	**(0.09, 0.63)**	**0.30**	**(0.20, 0.42)**	**0.31**	**(0.23, 0.37)**	**0.30**	**(0.27, 0.33)**
Pareto 0.95	0.32	(0.14, 0.55)	**0.30**	**(0.19, 0.43)**	**0.31**	**(0.22, 0.39)**	**0.30**	**(0.26, 0.34)**
Hybrid Empirical	0.32	(0.20, 0.48)	0.31	(0.21, 0.41)	**0.31**	**(0.24, 0.37)**	**0.30**	**(0.26, 0.34)**
Hybrid Pareto 0.9	**0.31**	**(0.09, 0.60)**	**0.30**	**(0.20, 0.42)**	**0.31**	**(0.23, 0.37)**	**0.30**	**(0.27, 0.33)**
Hybrid Pareto 0.95	0.32	(0.15, 0.54)	**0.30**	**(0.19, 0.43)**	**0.31**	**(0.22, 0.39)**	**0.30**	**(0.26, 0.34)**

Table S5. The mean and middle 95% (2.5% quantile, 97.5% quantile) of the Rogan-Gladen adjusted prevalence estimates when targeting a specificity of 0.95. The method(s) with the prevalence estimate nearest the truth in each scenario or equivalent after rounding are bolded.

	Scenario A				Scenario B			
	n=50		n=200		n=50		n=200	
Prevalence=0.05								
Empirical	**0.06**	**(0.00, 0.15)**	**0.05**	**(0.01, 0.10)**	0.07	(0.01, 0.15)	**0.05**	**(0.02, 0.10)**
Normal	0.07	(0.01, 0.14)	0.07	(0.03, 0.10)	0.06	(0.00, 0.16)	0.04	(0.00, 0.10)
Log Normal	0.08	(0.03, 0.13)	0.07	(0.04, 0.11)	0.08	(0.02, 0.16)	0.07	(0.03, 0.12)
MAD	0.15	(0.09, 0.25)	0.15	(0.10, 0.20)	0.21	(0.12, 0.29)	0.20	(0.15, 0.26)
Log MAD	0.11	(0.05, 0.20)	0.11	(0.07, 0.15)	0.16	(0.06, 0.26)	0.16	(0.11, 0.22)
Pareto 0.9	**0.06**	**(0.00, 0.13)**	**0.05**	**(0.02, 0.09)**	**0.05**	**(0.00, 0.14)**	0.03	(0.01, 0.08)
Hybrid Empirical	**0.06**	**(0.00, 0.14)**	**0.05**	**(0.01, 0.10)**	0.07	(0.01, 0.15)	**0.05**	**(0.02, 0.10)**
Hybrid Pareto 0.9	**0.06**	**(0.00, 0.13)**	**0.05**	**(0.02, 0.09)**	**0.05**	**(0.00, 0.14)**	0.03	(0.01, 0.08)
Prevalence=0.30								
Empirical	**0.31**	**(0.23, 0.38)**	**0.30**	**(0.26, 0.34)**	0.31	(0.27, 0.37)	**0.30**	**(0.28, 0.34)**
Normal	0.32	(0.26, 0.37)	0.31	(0.28, 0.34)	0.31	(0.25, 0.38)	0.29	(0.26, 0.34)
Log Normal	0.32	(0.27, 0.37)	0.32	(0.29, 0.35)	0.32	(0.27, 0.39)	0.31	(0.28, 0.35)
MAD	0.38	(0.32, 0.45)	0.37	(0.34, 0.41)	0.41	(0.35, 0.48)	0.41	(0.37, 0.46)
Log MAD	0.35	(0.30, 0.41)	0.34	(0.31, 0.38)	0.38	(0.31, 0.45)	0.38	(0.34, 0.43)
Pareto 0.9	**0.31**	**(0.23, 0.36)**	**0.30**	**(0.26, 0.33)**	**0.30**	**(0.26, 0.36)**	0.29	(0.26, 0.32)
Hybrid Empirical	**0.31**	**(0.23, 0.37)**	**0.30**	**(0.26, 0.34)**	0.31	(0.27, 0.38)	**0.30**	**(0.28, 0.34)**
Hybrid Pareto 0.9	**0.31**	**(0.23, 0.37)**	**0.30**	**(0.26, 0.33)**	**0.30**	**(0.26, 0.37)**	0.29	(0.26, 0.32)
